# Feasibility of Quantification of Intracranial Aneurysm Pulsation with 4D CTA with Manual and Computer-Aided Post-Processing

**DOI:** 10.1371/journal.pone.0166810

**Published:** 2016-11-23

**Authors:** Till Illies, Dennis Saering, Manabu Kinoshita, Toshiyuki Fujinaka, Maxim Bester, Jens Fiehler, Noriyuki Tomiyama, Yoshiyuki Watanabe

**Affiliations:** 1 Department of Diagnostic and Interventional Neuroradiology, University Medical Center Hamburg-Eppendorf, Hamburg, Germany; 2 Information Technology and Image Processing, University of Applied Sciences, Wedel, Germany; 3 Department of Neurosurgery, Osaka University Graduate School of Medicine, Osaka, Japan; 4 Department of Radiology, Osaka University Graduate School of Medicine, Osaka, Japan; Heinrich-Heine-Universitat Dusseldorf, GERMANY

## Abstract

**Background and Purpose:**

The analysis of the pulsation of unruptured intracranial aneurysms might improve the assessment of their stability and risk of rupture. Pulsations can easily be concealed due to the small movements of the aneurysm wall, making post-processing highly demanding. We hypothesized that the quantification of aneurysm pulsation is technically feasible and can be improved by computer-aided post-processing.

**Materials and Methods:**

Images of 14 cerebral aneurysms were acquired with an ECG-triggered 4D CTA. Aneurysms were post-processed manually and computer-aided on a 3D model. Volume curves and random noise-curves were compared with the arterial pulse wave and volume curves were compared between both post-processing modalities.

**Results:**

The aneurysm volume curves showed higher similarity with the pulse wave than the random curves (Hausdorff-distances 0.12 vs 0.25, p<0.01). Both post-processing methods did not differ in intra- (r = 0.45 vs r = 0.54, p>0.05) and inter-observer (r = 0.45 vs r = 0.54, p>0.05) reliability. Time needed for segmentation was significantly reduced in the computer-aided group (3.9 ± 1.8 min vs 20.8 ± 7.8 min, p<0.01).

**Conclusion:**

Our results show pulsatile changes in a subset of the studied aneurysms with the final prove of underlying volume changes remaining unsettled. Semi-automatic post-processing significantly reduces post-processing time but cannot yet replace manual segmentation.

## Introduction

Treatment strategies for patients with unruptured intracranial aneurysms are part of an ongoing debate. From large population based studies, there is strong evidence that the size and location of aneurysms influence their risk of rupture [1,2]. However, the correlation of size and bleeding risk is challenged by the finding of many small, ruptured aneurysms, showing the need to further refine risk prediction strategies. There is growing evidence for additional imaging findings which correlate with an elevated bleeding risk. Contrast uptake in the aneurysm wall is thought to represent areas of focal inflammation, which weakens the wall and renders the aneurysm unstable. Likewise, focal outpouchings of the aneurysm wall, i.e. daughter aneurysms, have been shown to correlate with an increased risk of rupture[3,4]. The analysis of the pulsation of cerebral aneurysms is a technique, which has been studied mostly visually-qualitatively. There are only few reports on the quantification of the aneurysm pulsation and this technique might give important objective and comparable information[5–7]. Conclusions regarding the composition and stability of the aneurysm wall might be drawn from the pulsations. The prediction of a future rupture might be improved and new insights given into the pathophysiologic processes involved in the aneurysm rupture [2]. The analysis is based on an ECG gated four-dimensional CT angiography (4DCTA) with high spatial resolution and a temporal resolution of 10 images in the cardiac cycle [5,7–12]. Volume changes of the cerebral vasculature and aneurysms within the cardiac cycle are very small. The wall excursions of a spherically shaped aneurysm of 5mm diameter can be estimated to be in the order of or below the resolution of the CTA [5,7], so that the image noise significantly interferes with the pulsation measurements. This raises concern that observed volume changes are due to noise rather than true pulsation. There is no study yet, which has approached the verification of the pulsation measurement. This is due to the fact that there is no appropriately tested vessel model for the pulsations in the magnitude of the cerebral vasculature as a gold standard. So, demonstration of the image’s quality can only be conducted by means of plausibility. The similarity of the time-course of the aneurysm’s volumes and the arterial pulse wave, which we used in this study, could serve as such an indicator for actual pulsation. Furthermore, the requirements for post-processing are high and imprecisions can easily cause inaccurate results or even conceal pulsation. Manual post-processing is time consuming and prone to error especially in the context of complex aneurysm shapes, which can be difficult to understand when presented in 2-dimensions. Specifically, it was shown that computer-aided segmentation significantly improves the otherwise limited intra- and inter-observer agreement of manual post-processing[13]. We therefore developed a software tool for the semi-automatic segmentation of cerebral aneurysms from the 4DCTA data. Additionally, the segmentation was performed on a three-dimensional model of the vasculature (3D+t model) in order to facilitate the understanding of the anatomy of the aneurysm and improve reproducibility. Both factors were intended to reduce post-processing time and increase accuracy, making the segmentation of these large datasets more robust and practically available. In this study, we hypothesized that the measurement of aneurysm pulsation is technically feasible and that the 3D computer assisted segmentation improves accuracy and reduces the time needed for segmentation compared to 2D manual segmentation.

## Material and Methods

### Patients

Twelve patients (8 male and 4 female, mean age of 59 years) with 14 unruptured cerebral aneurysms (2 internal carotid artery (ICA), 6 medial cerebral artery (MCA), 2 anterior communicating artery (AComA), 2 anterior choroidal artery (AChoA), 2 posterior communicating artery (PComA)) underwent 4DCTA (Table 1). The data were collected, anonymized and so provided by Y.W., Department of Neuroradiology, Osaka University Graduate School of Medicine, Osaka, Japan [5,6]. The research ethics committee of the University of Osaka evaluated and approved the use of the anonymized clinical data for this study and waived the requirement for written informed consent from patients.

**Table 1:** Aneurysm characteristics.

Aneurysm	Patient Age (Years)	Location (Artery)	Mean Diameter (mm)	Segmentation time (min)		Pulsation	
				2D	3D	2D	3D
1	51	MCA	17	31	4	1.04	1.04
2	70	AChA	6	29	1	1.06	1.02
3	69	PcomA	9	20	3		
4	50	ICA	15	21	4		
5	68	AChA	5	19	1	1.07	1.2
6	55	MCA	10	16	3		
7	55	MCA	7	18	5		1.2
8	66	MCA	9	15	7	1.04	
9	66	ACOM	6	15	2		
10	53	MCA	15	32	6		
11	49	ACOM	7	15	4		
12	43	PcomA	6	10	3		
13	71	MCA	6	15	5		
14	58	ICA	9	35	6		

Abbreviations for aneurysm location: ACOM anterior communicating artery, ACA anterior cerebral artery, AChA anterior choroidal artery, ICA internal carotid artery, MCA middle cerebral artery, PcomA posterior communicating artery. Mean aneurysm diameter in the axial plane. Time needed for 2D manual and 3D computer aided segmentation. Pulsation Group: 1 –with a pulsation like volume profile similar to the arterial pulse wave, 2 –with a randomly oscillating volume profile, 3 –with a predominantly flat volume profile.

### 4DCTA Acquisition

Retrospectively ECG-gated CTA was performed on a 320-detector Aquilion ONE CT (Toshiba, Nasu, Japan-). The following parameters were used: 120-kV tube voltage, 270-mA tube current, 350-ms gantry rotation time, 140-mm z-coverage. Contrast medium (Optiray 320 mgI/mL; Coviden Japan, Tokyo, Japan) was injected at 5-mL/s. Timing for the image acquisition was determined with a test injection of 15 mL contrast medium. For the CTA 50 mL of contrast medium were injected followed by a saline flush. 4DCTAs were reconstructed with half reconstruction using filtered back-projection and a kernel optimized for intracranial vessel imaging as well as Adaptive Iterative Dose Reduction 3D (AIDR 3D) with 10 steps of each 10% of the R-R interval, 512x512 image matrix, 0.5 mm slice thickness, 0.39 x 0.39 mm in plane resolution.

### Manual post-processing

All measurements were conducted by a neuroradiologist with 8 years of experience (M.B.). Post-processing was performed using the software Mevislab (Mevislab, Bremen, Germany), which provided axial, coronal and sagittal reconstructions of the vessels and segmentations. The vasculature was segmented from the cerebrospinal fluid with a threshold of 160 to 890 HU in order to minimize user interaction to the neck of the aneurysm as was done in previous studies [5,6]. The segmentation could be adjusted manually in case the threshold failed to separate the vasculature from the surrounding tissues. The aneurysm’s neck was then segmented manually in all time frames for all aneurysms. The time needed for the segmentation was recorded.

### Semi-automatic post-processing

**Fig 1:**
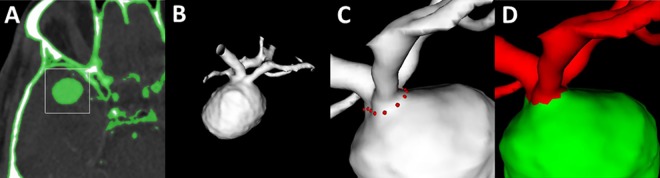
Workflow on the 3D+t model: On axial images the vasculature is defined with a threshold (green). To reduce computation time for the 3D+t model, the volume for post-processing is reduced by placing a VOI over the aneurysm (A). The resulting 3D+t model can be rotated, translated and zoomed (B). Segmentation points are placed on the aneurysms neck (C) and the aneurysm (green) is separated from the parent vessel (red) and the volume and surface is calculated (D).

All measurements were conducted by the same neuroradiologist with 8 years of experience (M.B.) with the in-house developed post-processing software AnToNIa (Analysis Tool for Neuroimaging Data) and a delay of three weeks [14–16]. As in the manual segmentation, the vasculature was segmented from the cerebrospinal fluid with a threshold of 160 to 890 HU. Based on this segmentation, a single surface model was generated for every time-point using the marching cube algorithm (3D+t model). The surface model can be rotated, moved and zoomed to enable the best view on relevant details. To segment the aneurysm from its parent vessel, segmentation points were manually placed on the 3D+t model at time point zero. These initial segmentation points were automatically transferred to all other time points using an iterative closest point approach and could be corrected manually (Fig 1). For each time point, the intraluminal segmentation plane was calculated by automatically connecting these manually placed segmentation points. The final segmentation was displayed on axial, coronal and sagittal planes. The time needed for segmentation was recorded.

### Statistical Analysis

R and RStudio 0.99 were used for statistical analysis. To quantify the probability that the volume curves are result of random image noise, the manual segmentation curves were compared with an arterial pulse wave curve of the middle cerebral artery derived from ultrasound examinations (S1 Table) [17]. For this purpose, the Hausdorff distances were calculated between the arterial pulse wave and the volume curves, which were normalized to a minimum of 0 and a maximum of 1, as well as between a set of randomly generated curves with the noise characteristics of the CT images (mean = 0.5, standard deviation = 0.2) and the pulse wave. For validation, the image noise was measured in an ROI placed in a homogenous region in the aneurysm of patient 1. The Hausdorff distances of the volume curves and the random curves were compared with a t-test [18].

For the comparison of both post-processing methods, the variability within each modality in terms of intra- and inter-observer agreements were explored with a Bland-Altman analysis and the intra-class correlation coefficient (ICC) [19]. An ICC greater than 0.75 was considered good agreement. The inter-modality agreement was analyzed with the Spearman correlation coefficient (SCC). The times needed for segmentation were compared using a two-sided t-test. P values less than 0.05 were assumed to indicate statistical significance.

## Results

### Patients and Aneurysms

Overview data of the patients, aneurysm characteristics, and segmentation times are provided in Table 1.

### Analysis of the temporal volume changes

**Fig 2:**
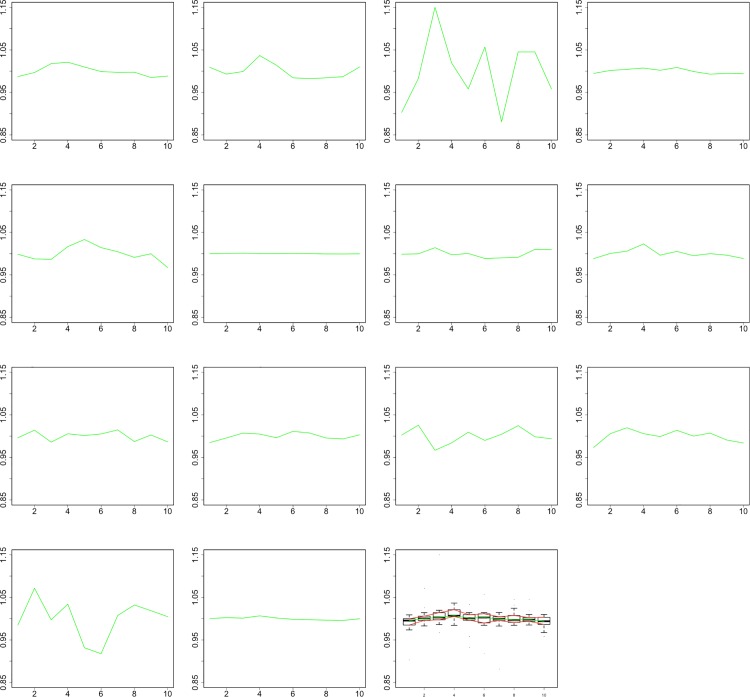
Relative volume changes of all 14 aneurysms with manual segmentation. Aneurysms 1, 2, 5, and 8 show a pulsation-like profile. Plot number 15 shows the cumulative pulsation of all aneurysms.

**Fig 3:**
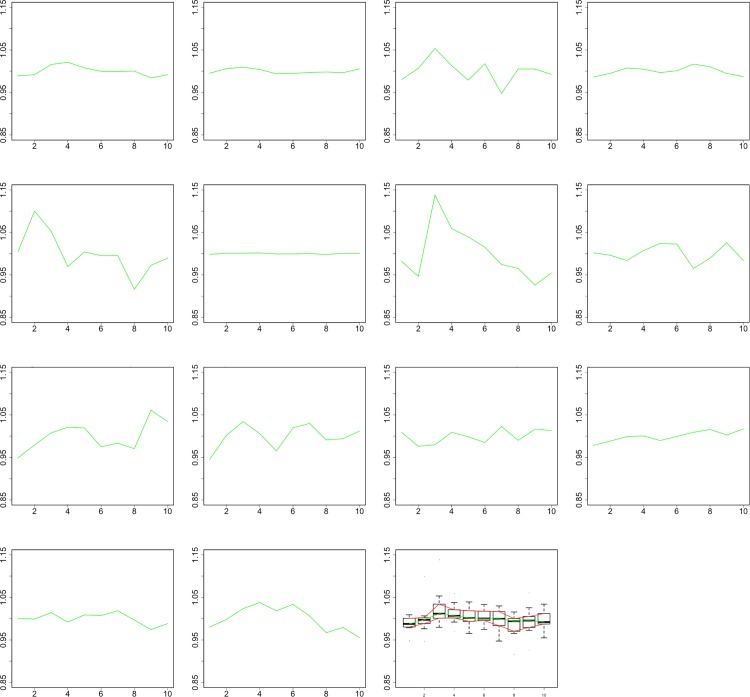
Relative volume changes of the studied aneurysms with semi-automatic segmentation. Aneurysms 1, 2, 5, and 7 show a pulsation-like profile. Plot number 15 shows the cumulative pulsation of all aneurysms.

**Fig 4:**
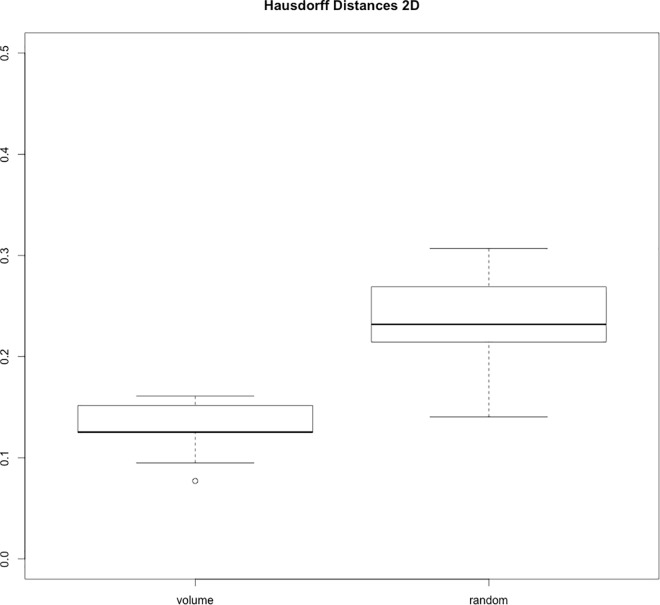
Boxplots of Hausdorff distances between the volume curves and random curves and the pulse wave on the right and left-hand side (0.12 vs 0.25, p<0.01).

Qualitative assessment of the volume curves, which were normalized to a mean of one to enable comparability, shows four curves with a pulsational profile in each post-processing group (aneurysms 1,2,5,8 for 2D and 1,2,5,7 for 3D+t, Table 1, Figs 2 and 3, S2 Table). The Hausdorff distances were significantly smaller between the volume curves and the arterial pulse profile compared to the distances between the random curve set and the arterial pulse profile (0.12 vs 0.25, p<0.01, S3 Table and Fig 4). Noise measurements in a homogeneous region in the aneurysm 1 showed a deviation of 18% (S4 Table).

### Intra- and interobserver and intermodality agreement

**Fig 5:**
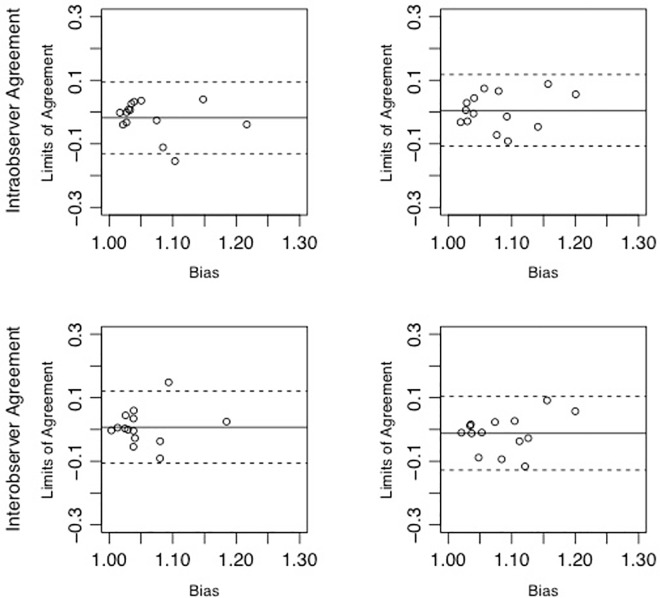
Results of the intraobserver (mean±LOA -0.02 ± 0.11 vs. 0.01 ± 0.11, upper row) and interobserver agreements (mean±LOA 0.007 ± 0.11 vs. -0.01 ± 0.12 lower row) for the 2D post-processing (right) and 3D+t post-processing (left).

Between 2D and 3D+t measurements, intraobserver (r = 0.61 vs r = 0.59) and interobserver agreements (r = 0.45 vs r = 0.54) did not differ. In the Bland-Altman analysis, the bias and limits-of-agreement were similar for intraobserver (-0.02 ± 0.11 vs. 0.01 ± 0.11) and inter-observer (0.007 ± 0.11 vs. -0.01 ± 0.12) agreements (Fig 5). There was moderate intermodality correlation of the volume pulsations (1.08 ± 0.05 vs 1.06 ± 0.05, p = 0.21 and r = 0.04).

### Time needed for segmentation

The segmentation time was significantly smaller for 3D than for 2D post-processing (3.9 ± 1.8 min vs 20.8 ± 7.8 min, p<0.001).

## Discussion

We studied the feasibility of the quantification of aneurysm pulsation with 4DCTA with manual and computer-assisted post-processing. Our results show pulsatile changes in a subset of the studied aneurysms but the prove of underlying volume inflation and deflation remains unsettled. Semi-automatic segmentation significantly reduces the post-processing with a similar reliability to manual post-processing.

As outlined in the introduction, 4DCTA pulsation measurements can be significantly falsified because the pulsation movements of the vessel wall are very small, in the order of or below the CT’s resolution. Therefore, assessing the methods feasibility at this preliminary stage is important to determine the standpoint in this research field and prompt further effort. Since there is no valid model of vessel pulsations of these dimensions, which could be used to validate the technique, this assessment has to be made by examining the method’s plausibility. In the presented study, we analyzed the similarity of the aneurysm’s volume profiles with the arterial pulse wave curve as an indication of pulsation. The formation of the arterial pulse wave by the systole and diastole and its transition through the cervical vasculature is the substrate for the pulsation of the cerebral vasculature. Hence, the volume profile of cerebral aneurysms during the cardiac cycle can be expected to follow the arterial pulse wave. A subset of the volume curves showed a pulsation-like profile with an early volume maximum corresponding to the systole and an otherwise smooth progression. To substantiate this qualitative assessment, we aimed to quantify the probability that the volume curves are randomly configured like the arterial pulsation profile. For this purpose, we compared the Hausdorff distances of the volume curves on the one hand and a set of randomly generated curves, with the characteristics of the image noise, on the other hand with the arterial pulse wave. The significantly smaller Hausdorff distances of the volume curves show that their shape is unlikely the result of random image noise and the curves contain pulsational information. Nevertheless, this does not prove that the pulsation is caused by inflation and deflation but could also be caused by translational and rotational movements. The overall movements of the vasculature are spatially inhomogeneous and the assignment of a specific quantity of inflation, deflation, translation or rotation to each voxel is not possible without taking model assumptions. Therefore, we could not correct for the possible error introduced by translation and rotation, as this requires further preliminary studies. Translation is a straight motion and occurring in the vasculature, results in the same amount of motion into the same direction of corresponding vessel wall sections. An adequately small translation would lead to changes in partial volume effects on both sides of the vessel section, which, by its definition, cancel each other out in volume calculations. The same is true for rotations. Otherwise, small rotations, as they can be assumed for the intracranial vasculature, can be estimated as translational movements with only tiny error due to the characteristics of the cosine function for small angles. Despite these theoretical considerations, it cannot be excluded that one or both introduce error into the pulsation measurement, which eventually has to be tested in an in-vitro model.

Furthermore, the results show a lack of reliability since only a subset of the aneurysms showed comprehensible pulsation-like volume profiles. The number of aneurysms with a pulsation-like volume profile is too low to draw further conclusions regarding a relationship of detectable pulsation and the aneurysm size or location. The most important reason for this is the interference of the high image noise with the tiny pulsation movements. It remains unclear whether in some aneurysms no pulsation can be detected because the pulsation movements are covered by image noise and therefore cannot be extracted or if imprecisions in post-processing make their detection impossible. It was the second objective of this study to approach the latter problem with the hypothesis that semi-automatic post-processing could improve the pulsation detection, as computer-aided segmentation is known to improve accuracy[13]. We utilized a simple thresh-holding to segment the vasculature including the aneurysms from the surrounding tissue for both, the manual and computer-aided post-processing, in order to reduce the manual input in the segmentation of the aneurysm to its neck. This approach might be a simplification, resulting in segmentation errors and masking pulsation. We are currently implementing a modified semi-automatic vessel segmentation based on a method, which we developed for TOF data in MRI, focusing it on the partial volume effects originating from the sub-voxel pulsation excursions[20,21]. This method has to be implemented and tested before it can be applied in future work. Our semi-automatic segmentation software was designed to facilitate orientation of the anatomy, reduce user interaction and accelerate the post-processing procedure. The software creates a three-dimensional model of the aneurysm and its parent vessel, which can be rotated freely, so complex anatomy can be comprehended more easily. This reduces handling time and is thought to improve correct and reproducible aneurysm segmentation. Moreover, for geometrical reasons, the segmentation error in a 3D volume is theoretically smaller than in a 2D plane. The aneurysm’s model is segmented in one time-point and the segmentation is then copied and adapted to the model’s surfaces in all time frames with the possibility to be corrected in each [22]. These features explain the significant reduction in post-processing time. Nevertheless, computer-aided post-processing did not show to be superior to manual segmentation in terms of observer reliability and quantity of pulsational-like profiles. A reason for the missing superiority, despite the theoretical advantages, might be found in the computation of the intravascular segmentation from the segmentation points on the model’s surfaces. This is calculated by a shape-based registration method and is subject to changes according to the pulsating aneurysm surface. This change can lead to registration errors resulting in an inaccurate transfer of the cutting-points, i.e. the intravascular segmentation is a function of the superficial segmentation. Small changes of the superficial segmentation could result in un-corresponding changes of the intravascular segmentation, leading to inaccurate volume calculations. To test the influence of different segmentation algorithms on the reliability of the intravascular segmentation is left for future work. This lack of accuracy could be controlled by increasing direct user control, for example a further manual segmentation step on 2D images. However, this will likely outweigh the timesaving and might introduce user-dependent error again. Contrary, a further automation with autonomous detection of the vessel wall and its interpolation at the aneurysm neck could improve this shortcoming. Several solutions with promising results of automatic aneurysm segmentations have been reported[23–28]. They have been tested on absolute volumes only and not pulsatilities, though, and their reliability needs to be evaluated for complex anatomies and wide-neck aneurysms. All of the above difficulties in computation of the intravascular segmentation might outweigh the advantages of automation in the 3D volume measurement, rendering its accuracy not superior to that of the 2D measurement.

For future work, a pulsatile vessel model needs to be established to be able to confirm the feasibility of aneurysm pulsation measurement, determine the accuracy of it and test and develop the processes involved in it. Moreover, these tests need to be performed on a much larger study group not least to account for the heterogeneous group of intracranial aneurysms. In the context of the presented argumentation we see our work as a preliminary study on future research on aneurysm pulsation quantification.

## Conclusion

Our results show pulsatile changes in a subset of the studied aneurysms. The prove of underlying volume changes remains unsettled so that further research is required before pulsation quantification could become applicable. Three-dimensional semi-automatic segmentation of cerebral aneurysms significantly reduces the post-processing time but cannot yet replace manual segmentation.

## Supporting Information

S1 Table Arterial pulsation in the middle cerebral artery. Arterial pulsation curve values for 10 time points used for the calculations [17].
(XLSX)

S2 Table Measured aneurysm volumes. Measurement data for manual segmentation (2D) and semi-automatic post-processing (3D) for all 14 aneurysms and 10 time-points of the cardiac cycle.
(XLSX)

S3 Table Hausdorff distances. Hausdorff distances between the arterial pulsation profile and the volume curves (upper row) of all aneurysms and the random curves (lower row).
(XLSX)

S4 Table Noise measurements in an exemplary aneurysm. ROI measurements in a homogeneous region in aneurysm 1 in all time points for validation of the image noise for calculation of the random curves.
(XLSX)
